# Functional Characterization of OTU Domain-Containing Deubiquitinases from Plant Pathogenic Fungi Reveals Distinct Immune Modulatory Mechanisms

**DOI:** 10.3390/jof12050361

**Published:** 2026-05-14

**Authors:** Sezer Akgöl, Serpil Aylin Yaşar, Fatih Kocabaş

**Affiliations:** 1 Medizinische Klinik und Poliklinik I, LMU University Hospital, 80336 Munich, Germany; sezer.akoel@med.uni-muenchen.de; 2 Department of Genetics and Bioengineering, Faculty of Engineering, Yeditepe University, İstanbul 34755, Türkiye; saylinyasar11@gmail.com; 3 Department of Molecular Biology and Genetics, Faculty of Engineering and Natural Sciences, Istanbul Atlas University, İstanbul 34403, Türkiye

**Keywords:** *Melampsora larici-populina*, *Taphrina deformans*, OTU domain, deubiquitinase, immune evasion, fungal plant pathogens

## Abstract

Ubiquitination is a key post-translational modification regulating cellular signaling and innate immunity, and its reversal by deubiquitinases (DUBs) represents a critical mechanism exploited by pathogens for immune evasion. While ovarian tumor (OTU) domain-containing DUBs are well characterized in viral systems, their roles in fungal pathogens remain largely unexplored. In this study, we investigated two putative OTU domain-containing proteins derived from the plant pathogenic fungi *Melampsora larici-populina* (MlpOTU, EGG09943.1) and *Taphrina deformans* (TdOTU, CCG84064.1). Recombinant MlpOTU and TdOTU proteins were successfully expressed and purified from *E. coli* and exhibited high solubility and proper folding. Functional analyses in HEK293T cells demonstrated that both proteins significantly reduce global ubiquitination levels, confirming their deubiquitinase activity in vivo. Despite this shared enzymatic function, the two proteins displayed markedly distinct effects on host immune gene expression. MlpOTU selectively suppressed key antiviral effectors, most notably *MX1*, suggesting a targeted immune evasion strategy. In contrast, TdOTU induced robust upregulation of multiple immune-related genes, including type I interferons, indicating a divergent role. Neither MlpOTU nor TdOTU triggered robust apoptosis, supporting their role as modulators of host signaling rather than cytotoxic effectors. Collectively, these findings provide the first functional evidence that fungal OTU domain-containing proteins act as active deubiquitinases and reveal distinct strategies by which plant pathogens may manipulate host immune responses. This study establishes fungal OTU domains as promising targets for antifungal intervention and broadens our understanding of cross-kingdom evasion mechanisms.

## 1. Introduction

Ubiquitination is a reversible post-translational modification that regulates numerous cellular processes, including protein degradation, signal transduction, and immune signaling [1,2]. The process occurs in three steps involving an activating enzyme (E1), a conjugating enzyme (E2), and a ligating enzyme (E3). Deubiquitinating enzymes (DUBs) reverse these modifications by removing ubiquitin from target proteins. Ubiquitin itself contains seven lysine residues that allow the formation of different chain types; polyubiquitin chains linked via Lys-48 primarily target proteins for proteasomal degradation, while other linkages serve non-proteolytic regulatory functions. Through these diverse chain topologies, ubiquitination acts as an intracellular signaling system that controls innate and adaptive immune responses [3].

The innate immune system constitutes the first line of defense against pathogens, including bacteria, fungi, protozoa, and viruses. It relies on pattern recognition receptors (PRRs) such as Toll-like receptors (TLRs) and retinoic acid-inducible gene I (RIG-I)-like receptors (RLRs) to detect pathogen-associated molecular patterns (PAMPs) like viral genomic DNA, single- or double-stranded RNA, and viral proteins. PRR activation triggers the production of cytokines, particularly type I interferons (IFN-α/β), which induce the expression of interferon-stimulated genes (ISGs). Among these, ISG15, an ubiquitin-like protein, plays a critical role in establishing an antiviral state. Additionally, tumor necrosis factor-α (TNF-α) activates the transcription factor NF-κB by promoting phosphorylation and ubiquitin-dependent degradation of its inhibitor IκB, leading to the expression of pro-inflammatory and antiviral genes [4].

DUBs are classified into several families, among which the ovarian tumor (OTU) domain family has recently gained attention. OTU domains are present not only in eukaryotes but also in certain RNA viruses. For example, viruses of the Bunyaviridae family (genus Nairovirus) encode an OTU domain within their large (L) protein. Crimean-Congo hemorrhagic fever virus (CCHFV) and Dugbe virus (DUGV) use this domain to antagonize host innate immunity by deconjugating ubiquitin and ISG15 from host proteins [5,6,7]. CCHFV OTU exhibits both deubiquitinase and deISGylase activities, which suppress NF-κB and type I interferon pathways, key components of the antiviral and antimalarial response [8,9,10]. The structural basis of this inhibition has been characterized, and the OTU inhibition pocket is considered a promising target for antiviral drug development.

Interestingly, plant pathogens also produce effector molecules that manipulate host immune responses to promote infection. *Melampsora larici-populina* is a rust fungus that infects poplar leaves, causing severe damage to poplar plantations. *Taphrina deformans* is another fungal pathogen that induces leaf curl in peach trees, leading to substantial agricultural losses. Bioinformatics observations have revealed that certain proteins produced by these two fungi share sequence similarity with the CCHFV OTU domain, raising the possibility that they may employ similar immune evasion strategies.

In this study, we hypothesized that the putative OTU domains from *M. larici-populina* (MlpOTU) and *T. deformans* (TdOTU) function as genuine deubiquitinating enzymes that modulate host immune responses. To test this hypothesis, we expressed and purified recombinant MlpOTU and TdOTU proteins, characterized their solubility and folding, investigated their effects on host cellular ubiquitination and immune gene expression, and assessed their potential to induce programmed cell death. Our results provide the first functional characterization of these fungal OTU domains and establish them as potential targets for antifungal drug development.

## 2. Materials and Methods

### 2.1. Bacterial Expression Vectors and Cloning

The coding sequences for MlpOTU and TdOTU were obtained from the NCBI protein database. To enable efficient expression in *E. coli*, codon optimization was carried out using the OPTIMIZER web server (http://genomes.urv.es/OPTIMIZER/, accessed on 14 May 2018) with the *E. coli* K12 strain settings, following previously established protocols [8]. The codon usage table for highly expressed genes (HEG) was chosen, and the guided-random optimization strategy was applied. Each optimized nucleotide sequence was supplemented with a C-terminal 6×HisTag (CATCATCACCATCATCAC) and a stop codon (TAA) using the ApE plasmid editor. In-Fusion cloning-compatible homology arms (15–20 nucleotides) were appended to facilitate insertion into the pET-26b(+) vector, after verifying the absence of internal *NdeI*, *NcoI*, *XbaI*, and *EcoRI* sites. The final gene fragments were synthesized as gBlocks^®^ (IDT, Coralville, IA, USA) and subcloned into the pTwist31_Amp vector.

To construct the bacterial expression plasmids, the pTwist31_Amp vectors harboring the optimized MlpOTU (1056 bp) and TdOTU (1104 bp) inserts were subjected to digestion with *NdeI* and *NcoI* (New England Biolabs, Ipswich, MA, USA). The released fragments were separated by agarose gel electrophoresis, excised, and purified using a Qiagen gel extraction kit (Qiagen, Hilden, Germany). Concurrently, the pET-26b(+) vector (Novagen, Madison, WI, USA) was linearized with the same two restriction enzymes. Each insert was then ligated into the linearized pET-26b(+) backbone via the In-Fusion^®^ HD Cloning System (Clontech, Mountain View, CA, USA). The resulting recombinant plasmids, designated pET-26b-MlpOTU and pET-26b-TdOTU, were introduced into Stellar competent *E. coli* cells. Positive transformants were identified by colony PCR, and the correct constructs were verified by restriction mapping (*NdeI*/*EcoRI*) and DNA sequencing (Medsantek, Istanbul, Turkey). Table 1 summarizes the key properties of MlpOTU and TdOTU.

### 2.2. Chromatography, SDS-PAGE and Ultrafiltration

For protein expression, pET26b-MlpOTU and pET26b-TdOTU were transformed into *E. coli* BL21(DE3) competent cells. Single colonies were inoculated into 5 mL LB broth containing 50 μg/mL kanamycin and incubated overnight at 37 °C with shaking at 180 rpm. The overnight cultures were diluted 1:40 into 20 mL fresh LB broth with kanamycin and grown at 37 °C, 180 rpm until the optical density at 600 nm (OD_600_) reached 0.6. Protein expression was induced by adding 1 mM IPTG (1:1000 dilution), and the cultures were incubated overnight at 24 °C with shaking at 180 rpm. Cells were harvested by centrifugation at 11,000× *g* for 30 s. Cell pellets were resuspended in lysis buffer (8 M urea, 20 mM Tris-HCl, pH 8.0, 500 mM NaCl, 20 mM imidazole) and lysed by sonication (5 cycles of 30 s on ice). The lysate was centrifuged at 10,000× *g* for 30 min at 4 °C. Purification was performed using HisTrap HP affinity columns (GE Healthcare, Chicago, IL, USA) on an ÄKTAprime system. Bound proteins were eluted with an elution buffer (20 mM Tris-HCl, pH 8.0, 500 mM NaCl, 500 mM imidazole). Eluted fractions were pooled and concentrated using Amicon Ultra-0.5 mL centrifugal filters (30K NMWL, Millipore, Burlington, MA, USA) and buffer-exchanged into PBS (pH 7.4).

### 2.3. Solubility Analysis

Protein concentrations of purified MlpOTU and TdOTU were determined using the bicinchoninic acid (BCA) assay (Pierce™ BCA Protein Assay Kit, Thermo Scientific, Waltham, MA, USA) according to the manufacturer’s protocol. Proper protein folding was then assessed by solubility assay. Briefly, 25 μL of purified MlpOTU or TdOTU was added to 475 μL of phosphate-buffered saline (PBS, pH 7.4) and incubated for 1 h at 4 °C. Following incubation, samples were centrifuged at maximum speed (approximately 14,000× *g*) for 5 min. The supernatant (475 μL), containing properly folded soluble protein, was transferred to a new tube. The remaining pellet (25 μL), containing misfolded or aggregated protein, was resuspended in 75 μL of PBS. Protein concentrations in both supernatant and pellet fractions were measured using a NanoDrop 2000 spectrophotometer (Thermo Fisher Scientific Inc, Waltham, MA, USA) at A280 nm. Solubility factor was calculated as: [supernatant protein]/[supernatant + pellet protein] × 100. For SDS-PAGE analysis, samples were mixed with 4× Laemmli buffer, boiled for 5 min, and resolved on 12% polyacrylamide gels.

### 2.4. SDS-PAGE and Western Blot Analysis

Protein samples were mixed with distilled water (150 µL) and 4× Laemmli sample buffer (50 µL) to a final 1× concentration, then heated at 95 °C for 5 min and placed on ice. Samples were resolved on 12% SDS-polyacrylamide gels. After electrophoresis, gels were stained with Coomassie Brilliant Blue R-250 overnight on a shaker, then destained until the background became clear. Gel images were acquired using a gel documentation system. For Western blot analysis, proteins separated by SDS-PAGE were transferred to PVDF membranes. Membranes were blocked with 5% (*w*/*v*) non-fat dry milk in TBST (Tris-buffered saline with 0.1% Tween-20) for 1 h at room temperature. The membranes were then incubated overnight at 4 °C with mouse anti-HisTag primary antibody (His-Tag (27E8) mouse monoclonal, 1:1000 dilution in blocking buffer). After washing three times with TBST, membranes were incubated with HRP-conjugated anti-mouse secondary antibody (1:2000 dilution) for 1 h at room temperature. Following three additional washes with TBST, signal was detected using enhanced chemiluminescence (ECL) reagent and visualized with a Bio-Rad ChemiDoc XRS+ system (Bio-Rad Laboratories, Hercules, CA, USA).

### 2.5. Mammalian Expression Vector Construction

For mammalian expression studies, MlpOTU and TdOTU coding sequences were subcloned from pET26b(+) into the pcDNA3.1(+) mammalian expression vector (Invitrogen, Waltham, MA, USA). The pET26b-MlpOTU and pET26b-TdOTU plasmids were digested with restriction enzymes *EcoRI* and *NheI* (the latter compatible with *XbaI*) in a reaction incubated at 37 °C for 40 min, followed by enzyme inactivation at 65 °C for 20 min. The pcDNA3.1(+) vector was digested with *EcoRI* and *NdeI* overnight at 37 °C (or alternatively with *NheI* and *EcoRI* for compatibility; both strategies yield compatible ends). All digests were resolved on 1% agarose gels, and the desired DNA fragments (inserts and linearized vector) were excised and purified using a NucleoSpin Gel and PCR Clean-up kit (Takara Bio, Otsu, Shiga, Japan). Ligation reactions were set up with a vector:insert molar ratio of 1:3 using T4 DNA ligase and T4 ligase buffer. The reaction was incubated at room temperature for 40 min, then inactivated at 65 °C for 40 min. The ligation mixture (7 µL) was added to 100 µL of competent *E. coli* DH5α cells and incubated on ice for 30 min. Heat shock was performed at 42 °C for 90 s, followed by 2 min on ice. LB broth was added to a final volume of 1 mL, and the cells were incubated at 37 °C for 1 h. The culture was centrifuged at 1000 rpm for 5 min, 700 µL of supernatant was discarded, and the pellet was resuspended in the remaining supernatant. Aliquots (100 µL) were plated onto LB-agar plates containing 100 µg/mL ampicillin and incubated overnight at 37 °C. Single colonies were selected and grown in LB broth overnight. Recombinant plasmids were isolated using a NucleoSpin Plasmid kit (high-copy) (Takara Bio, Otsu, Shiga, Japan). Correct clones were confirmed by restriction digestion with *NheI* and *EcoRI* (or *EcoRI* and *NdeI*) and by DNA sequencing. Positive clones were then amplified using a NucleoBond Xtra Midi Plus kit (Takara Bio, Otsu, Shiga, Japan) for large-scale plasmid preparation. The integrity of the final constructs was verified again by restriction digestion (double digest with *EcoRI* and *NdeI*) and agarose gel electrophoresis alongside uncut plasmids.

### 2.6. Cell Culture and Transfection

HEK293T cells (ATCC^®^ CRL-3216™) were cultured in DMEM high glucose supplemented with 10% fetal bovine serum (FBS), 100 U/mL penicillin, and 100 μg/mL streptomycin at 37 °C in a humidified atmosphere of 5% CO_2_ and 95% relative humidity. For transfection, 3 × 10^5^ cells were seeded into 6-well plates with 2 mL of complete medium and incubated overnight. The next day, 1 μg of pcDNA3.1-MlpOTU, pcDNA3.1-TdOTU, or empty pcDNA3.1 vector (control) was transferred to separate 1.5 mL Eppendorf tubes, and 200 μL of serum-free DMEM was added to each tube. Then, 2 μg of polyethylenimine (PEI, 1 mg/mL stock) was added, and the mixture was mixed by pipetting. The plasmid–PEI complexes were incubated at room temperature for 15 min. The entire mixture was then added dropwise onto the HEK293T cells in the 6-well plate. Four hours post-transfection, the medium was removed and replaced with fresh complete DMEM. GFP expression (from a co-transfected or separate GFP control plasmid) was checked 16–24 h later under a fluorescence microscope to verify transfection efficiency. Cells were then returned to the incubator and harvested 72 h post-transfection for downstream analyses.

### 2.7. RNA Isolation and Real-Time PCR (Q-PCR)

Total RNA was isolated from transfected HEK293T cells using NucleoZOL reagent (Macherey-Nagel, Düren, Germany). The culture medium was discarded, and 500 μL of NucleoZOL was added per well. The solution was homogenized, collected, and transferred to a 1.5 mL tube. Then, 200 μL of DEPC-treated water per 500 μL of NucleoZOL was added, and the samples were vortexed for 15 s, followed by incubation at room temperature for 5 min. The lysate was centrifuged at 12,000× *g* for 15 min. The supernatant (500 μL) was transferred to a fresh tube, and 500 μL of isopropanol was added. After incubation at room temperature for 10 min, the mixture was centrifuged at 12,000× *g* for 10 min. The supernatant was discarded, and the pellet was washed with 75% ethanol (vortexed briefly) and centrifuged at 8000× *g* for 3 min. The ethanol wash was repeated once. The final pellet was dissolved in DEPC-treated water and vortexed for 3 min. RNA concentration and purity were determined by spectrophotometry. First-strand cDNA was synthesized from 750 ng of total RNA using random primers and reverse transcriptase (ProtoScript II First Strand cDNA Synthesis Kit, NEB, Ipswich, MA, USA). For each sample, the reaction mix (13 μL reaction mix, 2 μL random primers, 1 μL enzyme, and RNA adjusted to 750 ng with DEPC water) was incubated according to the manufacturer’s protocol. Real-time PCR (qPCR) was performed using SYBR Green Master Mix on a thermocycler (LightCycler 96, Roche, Basel, Switzerland). Each reaction contained: 6.5 μL distilled water, 2 μL cDNA template, 0.75 μL each of forward and reverse primers (10 μM), and 10 μL SYBR Green Master Mix (containing MgCl_2_, dNTPs, and DNA polymerase). Primers for target genes are listed in Table S1. The thermal cycling conditions were: 95 °C for 2 min, followed by 55 cycles of 95 °C for 15 s, 60 °C for 60 s, and 72 °C for 45 s. GAPDH was used as an internal control. Relative gene expression was calculated using the ΔΔCt method and normalized to the empty pcDNA3.1 vector control.

### 2.8. Protein Isolation and Western Blot for Ubiquitination Analysis

Protein isolation was performed using HEK293T cells transfected with pcDNA3.1 (empty vector control), pcDNA3.1-MlpOTU, or pcDNA3.1-TdOTU. Cells were first washed with ice-cold phosphate-buffered saline (PBS). Then, 500 μL of ice-cold 1× RIPA buffer (supplemented with protease inhibitors) was added to the cells, and the solution was homogenously distributed by pipetting. Cells were scraped from the culture surface and transferred into a 1.5 mL Eppendorf tube. The lysates were incubated at 4 °C with constant agitation for 30 min, followed by centrifugation at 12,000× *g* for 20 min at 4 °C. The supernatants (containing total protein) were collected and transferred to fresh tubes. Protein concentrations were determined using the Pierce™ BCA Protein Assay Kit (Thermo Scientific) according to the manufacturer’s protocol. After concentration determination, equal amounts of protein (adjusted volume) were mixed with 10 μL of 4× Laemmli buffer, heated at 95 °C for 5 min, and then placed on ice. Samples (20 μL) were resolved on 12% SDS-polyacrylamide gels (stacking gel 4%) at 70 V for 10 min followed by 100 V for 60 min. Proteins were transferred to polyvinylidene difluoride (PVDF) membranes using a semi-dry transfer system for 24 min. The membranes were blocked with 1% bovine serum albumin (BSA) in Tris-buffered saline (TBS) for 1 h at room temperature with constant agitation. After blocking, membranes were washed three times (5 min each) with TBS containing 0.1% Tween-20 (TBST). The membranes were then incubated overnight at 4 °C with primary antibody diluted 1:1000 in 1% BSA/TBST: either anti-mono/poly-ubiquitin antibody (FK2) or anti-poly-ubiquitin antibody (FK1). After three washes with TBST (5 min each), membranes were incubated with HRP-conjugated anti-mouse secondary antibody (1:2000 dilution in 1% BSA/TBST) for 1 h at room temperature with agitation. Membranes were washed again three times with TBST, and protein bands were visualized using a Bio-Rad ChemiDoc XRS+ imaging system with enhanced chemiluminescence (ECL) substrate. Beta-actin was used as a loading control and detected with a specific primary antibody followed by the same secondary antibody.

### 2.9. Apoptosis Analysis

Apoptosis was assessed using Annexin V-FITC/PI staining. The 4× binding buffer was diluted to 1× with distilled water. HEK293T cells transfected with empty pcDNA3.1 (control), pcDNA3.1-MlpOTU, or pcDNA3.1-TdOTU were washed with phosphate-buffered saline (PBS) and then resuspended in 200 μL of 1× binding buffer. An aliquot of 195 μL of the cell suspension was transferred to a new tube and mixed with 5 μL of Annexin V-FITC. The mixture was incubated at room temperature for 10 min in the dark. After incubation, the cells were washed with 200 μL of 1× binding buffer and then resuspended in 190 μL of fresh 1× binding buffer. Subsequently, 10 μL of propidium iodide (PI) was added. Samples were analyzed immediately by flow cytometry (Beckman Coulter CytoFLEX, Beckman Coulter, Pasadena, CA, USA).

### 2.10. Statistical Analysis

All experiments were performed at least in triplicate. Data are presented as mean ± SD. Statistical significance was determined using Student’s *t*-test. A *p*-value < 0.05 was considered statistically significant (* *p* < 0.05, ** *p* < 0.01). Student’s *t*-test was not applied for multi-group comparisons.

## 3. Results

### 3.1. Expression and Purification of Recombinant MlpOTU and TdOTU

**Figure 1:**
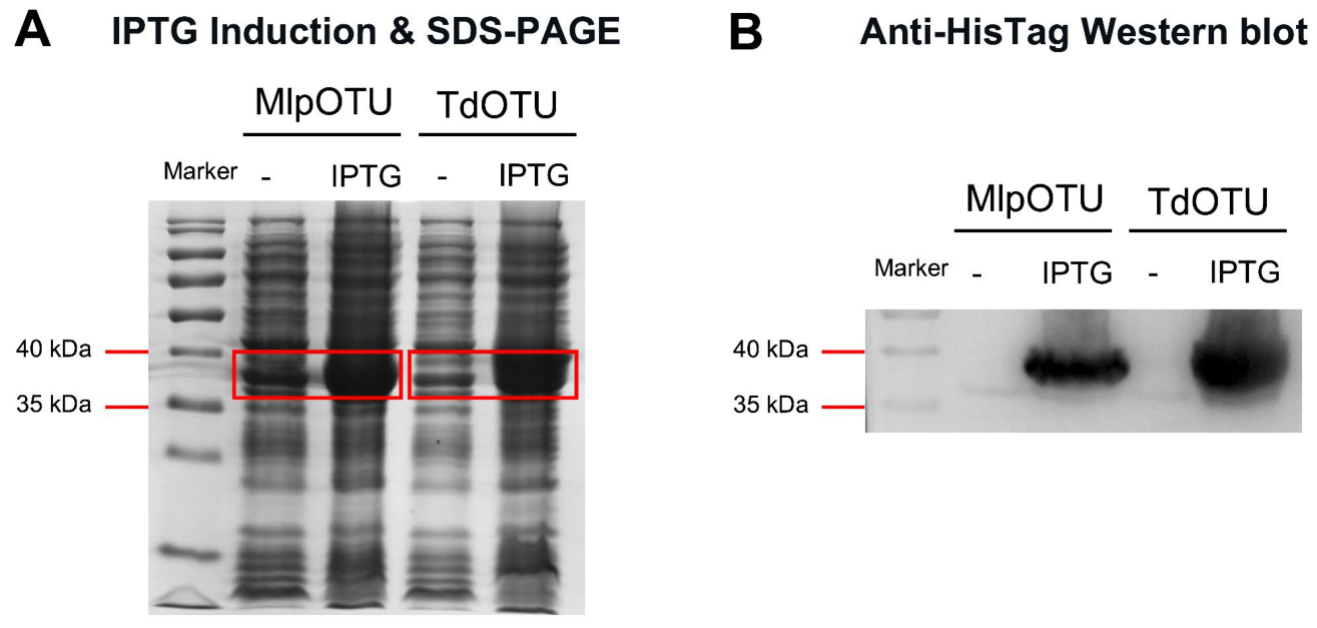
**Recombinant expression of MlpOTU and TdOTU proteins.** (**A**) SDS-PAGE analysis of MlpOTU (**left**) and TdOTU (**right**) expression in *E. coli* BL21(DE3) cells. Lanes: 1, uninduced control; 2, IPTG-induced. Red lines indicate expected bands at ~36 kDa (MlpOTU) and ~38 kDa (TdOTU). (**B**) Anti-HisTag Western blot confirmation of recombinant MlpOTU and TdOTU expression. Uninduced samples show faint bands, while IPTG-induced samples show strong specific signals at the expected molecular weights.

To obtain recombinant MlpOTU and TdOTU proteins, *E. coli* BL21(DE3) cells transformed with pET26b-MlpOTU or pET26b-TdOTU were induced with IPTG. SDS-PAGE analysis revealed prominent bands at approximately 36 kDa (MlpOTU) and 38 kDa (TdOTU) in induced lysates, which were absent in uninduced controls (Figure 1A). For both proteins, two concentrated bands appeared after IPTG addition, indicating successful induction. Western blot analysis using anti-HisTag antibody confirmed the identity of both recombinant proteins, with specific signals at the expected sizes and with no bands in uninduced samples (Figure 1B).

**Figure 2:**
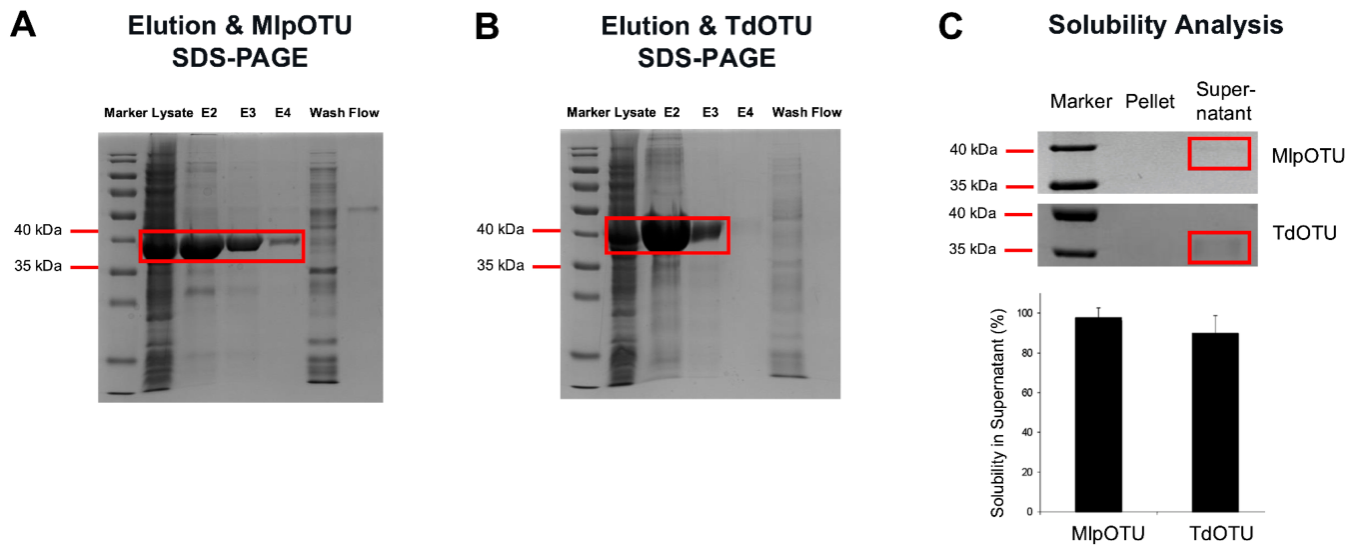
**Purification and solubility analysis.** (**A**) SDS-PAGE analysis of MlpOTU purification fractions including lysate, flow-through, wash, and elution fractions (E2, E3, E4) from HisTrap HP affinity chromatography. (**B**) SDS-PAGE analysis of TdOTU purification fractions under the same conditions. (**C**) Solubility assay of MlpOTU and TdOTU showing supernatant and pellet fractions and their quantification of solubility in supernatant. Both MlpOTU and TdOTU exhibited high solubility (>90%), indicating proper protein folding. Data are presented as mean ± SD.

Following affinity purification using HisTrap HP columns, SDS-PAGE analysis showed highly purified MlpOTU and TdOTU (Figure 2A,B). For MlpOTU, elutions 2, 3 and 4 contained the highest protein concentration; comparison with the crude lysate, wash and flow-through fractions demonstrated that most impurities were removed, although the elutions were not completely pure. Similarly, for TdOTU, elutions 2–3 showed marked enrichment of the target band, with no protein detected in wash or flow-through fractions. Both proteins were obtained at >90% homogeneity.

### 3.2. Solubility and Folding Analysis of Recombinant OTUs

Solubility analysis in PBS revealed that both MlpOTU and TdOTU exhibited high solubility, with 90% of purified protein remaining in the supernatant fraction after centrifugation (Figure 2C). SDS-PAGE analysis of MlpOTU showed a faint but visible band in the supernatant, while the pellet yielded no detectable band, indicating that a fraction of the protein was soluble. For TdOTU, a slightly clear band was observed in the supernatant, with no band in the pellet. Solubility percentages calculated from NanoDrop measurements confirmed high solubility for both proteins. Protein concentrations determined by BCA assay were 0.22 mg/mL for MlpOTU and 2.70 mg/mL for TdOTU (Table S2). The lower concentration of MlpOTU may be due to protein loss during ultrafiltration with 30 kDa filters.

### 3.3. MlpOTU and TdOTU Modulate Host Immune Gene Expression

To determine whether MlpOTU and TdOTU affect host innate immune responses, we performed Q-PCR analysis on HEK293T cells transfected with pcDNA3.1 (control), MlpOTU, or TdOTU. Expression levels of genes involved in pyroptosis, nucleic acid sensing, NF-κB signaling, and interferon responses were examined.

**Figure 3:**
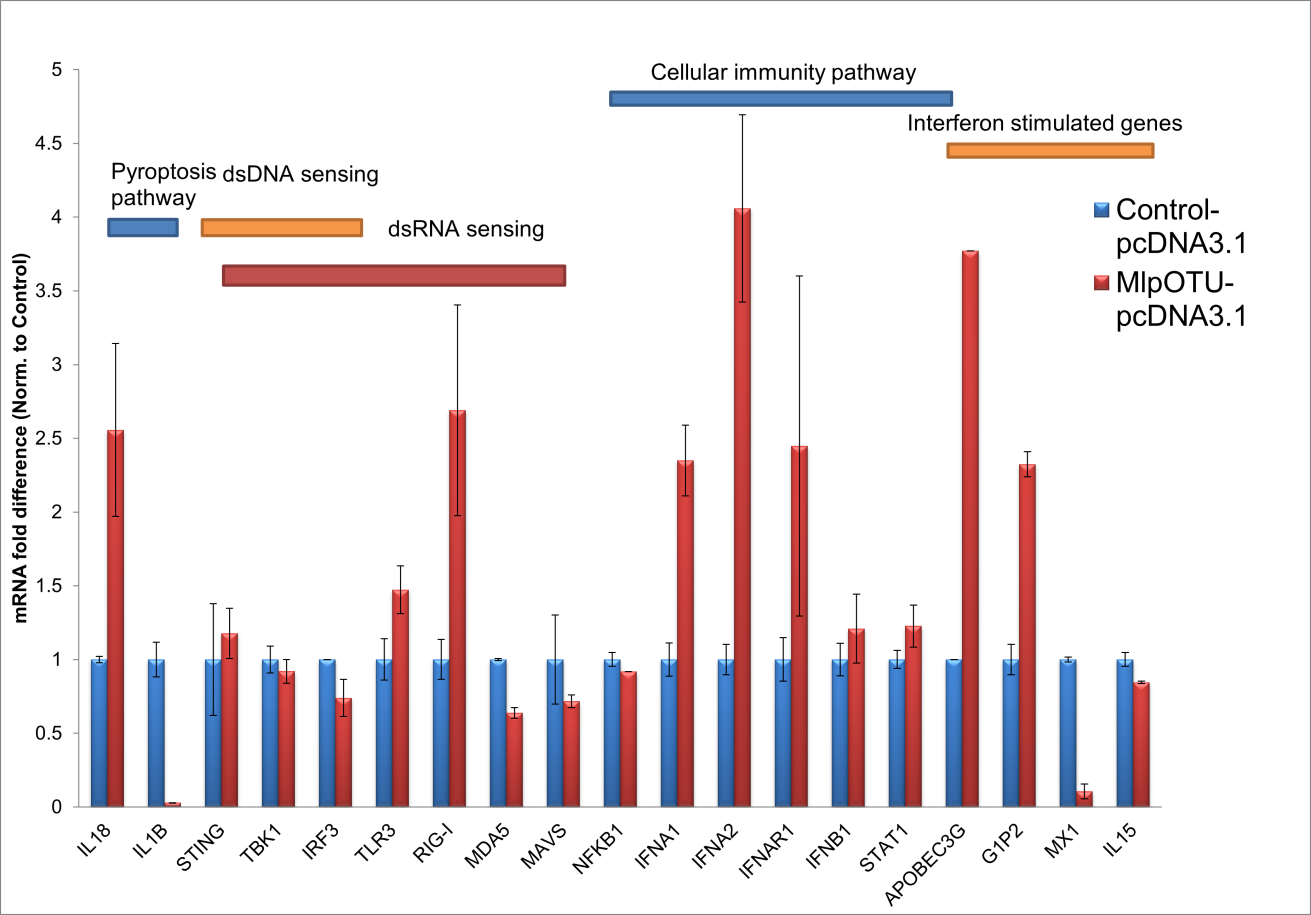
**Analysis of gene expression post MlpOTU-pcDNA3.1 treatment.** RT-PCR analysis of immune-related gene expression in HEK293T cells transfected with MlpOTU-pcDNA3.1 compared to empty pcDNA3.1 vector control (set to 1). Genes involved in pyroptosis (*IL18*, *IL1B*), DNA sensing (*STING*, *TBK1*, *IRF3*), RNA sensing (*TLR3*, *RIG-I*, *MDA5*, *MAVS*), NF-κB signaling (*NFKB1*), type I interferon response (*IFNA1*, *IFNA2*, *IFNB1*, *IFNAR1*, *STAT1*), and interferon-stimulated genes (*APOBEC3G*, *G1P2*, *MX1*, *IL15*) are shown. Data represent fold change relative to control. Data are presented as mean ± SD.

MlpOTU expression resulted in differential modulation (Figure 3). Pyroptosis-related genes showed mixed responses: *IL18* was increased, while IL1B was decreased. Among DNA sensing genes, *STING* and *TBK1* were unchanged, whereas IRF3 was slightly downregulated. RNA sensing genes showed increased *TLR3* and *RIG-I* but decreased *MDA5* and *MAVS* expression. Type I interferon genes (*IFNA1*, *IFNA2*, *IFNR1*) were upregulated, while the interferon-stimulated gene *MX1* was severely decreased. *APOBEC3G* and *G1P2* showed increased expression.

**Figure 4:**
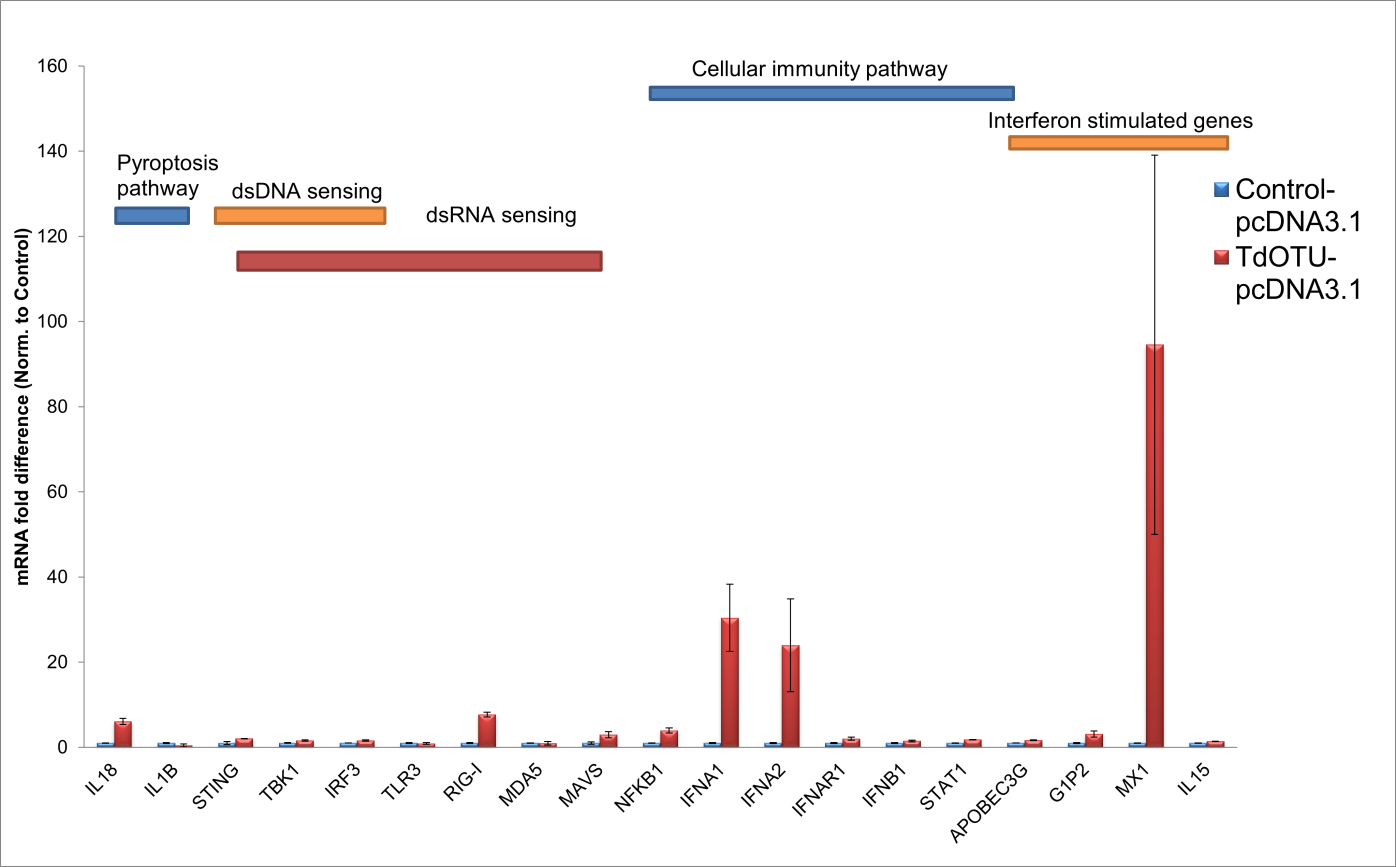
**Analysis of gene expression post TdOTU-pcDNA3.1 treatment.** RT-PCR analysis of the same immune-related gene panel in HEK293T cells transfected with TdOTU-pcDNA3.1 compared to empty pcDNA3.1 vector control (set to 1). TdOTU expression resulted in overexpression of most tested genes, with particularly strong increases in *IFNA1*, *IFNA2*, and *MX1*. Data represent fold change relative to control. Data are presented as mean ± SD.

In contrast, TdOTU expression led to major changes in several of tested genes (Figure 4). Particularly striking increases (over 20- to 100-fold) were observed for *IFNA1*, *IFNA2*, and *MX1*. *IL18*, *RIG-I*, *MAVS*, *NFKB1*, and *G1P2* showed moderate increases (2- to 4-fold). These results suggest that while MlpOTU may interfere with specific immune pathways (particularly MX1-mediated responses), TdOTU does not suppress host immunity through the tested genes.

### 3.4. MlpOTU and TdOTU Reduce Global Ubiquitination Levels

To evaluate the deubiquitinase activity of MlpOTU and TdOTU in a cellular context, whole-cell lysates from transfected cells were analyzed by Western blotting using antibodies recognizing either mono- and poly-ubiquitin chains (Mono & Poly UB) or poly-ubiquitin chains only (Poly UB). Empty vector-transfected cells served as a control.

**Figure 5:**
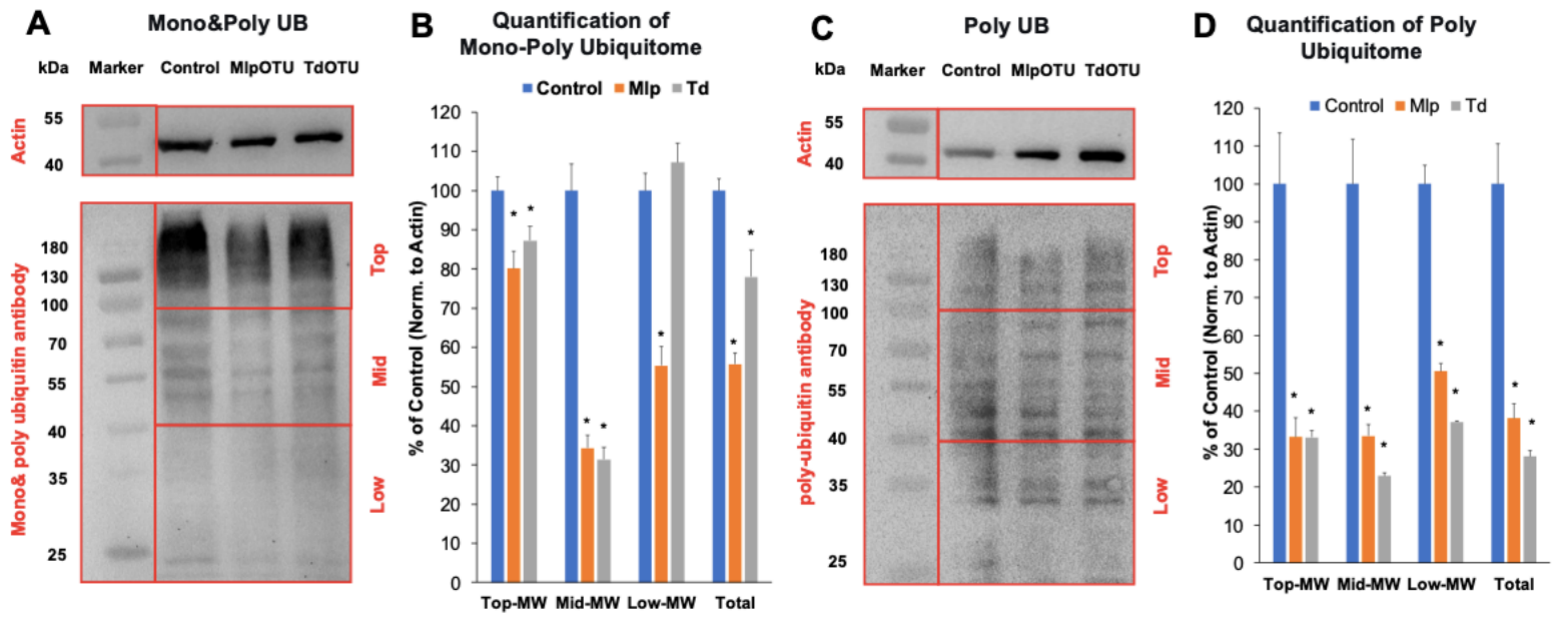
**Western blot analysis of ubiquitome.** (**A**) Representative Western blot of HEK293T cell lysates using mono- and poly-ubiquitin antibody. Beta-actin is shown as a loading control. (**B**) Densitometric quantification of mono-poly ubiquitin blots for top, middle and low molecular weight (MW) proteins as well as total. (**C**) Representative Western blot using poly-ubiquitin-specific antibody with beta-actin loading control. (**D**) Densitometric quantification of poly ubiquitin blots for top, middle and low molecular weight proteins as well as total. Densitometric quantification of blots showing reduced ubiquitination levels in MlpOTU- and TdOTU-expressing cells compared to empty vector control. Data are presented as mean ± SD. * *p* < 0.01.

In control cells, the Mono & Poly UB antibody detected a strong ubiquitin signal across a wide molecular weight range, including a high molecular weight smear, several discrete mid-range bands, and lower molecular weight species (Figure 5A). In cells expressing either MlpOTU or TdOTU, the overall ubiquitin signal intensity was markedly reduced. The decrease was most evident in the high molecular weight region and among the smallest ubiquitinated species, while some mid-range bands also showed diminished intensity (Figure 5B). Expression of MlpOTU reduced total Mono & Poly UB signals to 5568% of control levels, and TdOTU to 7849% (*p* < 0.01). Mono- and polyubiquitin signals were markedly reduced, particularly within the mid-molecular-weight range (40–100 kDa), in both MlpOTU and TdOTU. In this region, ubiquitin signal intensity decreased to 34% and 31% of control levels, respectively (*p* < 0.01). A similar reduction in ubiquitin signal was observed using the Poly UB antibody, which specifically detects poly-ubiquitin chains (Figure 5C). Notably, the reduction in poly-ubiquitin signal appeared more uniform across the entire molecular weight range compared to the Mono & Poly UB blots. For Poly UB-specific signals, MlpOTU reduced the signal to 38%, and TdOTU to 28% of control levels (*p* < 0.01) (Figure 5D). In contrast, polyubiquitin signals were markedly reduced in both MlpOTU and TdOTU, particularly across the mid- and high-molecular-weight protein ranges (>40 kDa), reaching as low as 22.9% of control levels (*p* < 0.01). Overall, both OTU proteins consistently reduced ubiquitin signals by 22–72% relative to the empty vector control. These quantitative data confirm that both MlpOTU and TdOTU possess functional deubiquitinase activity in vivo, with TdOTU exhibiting marginally higher apparent activity under the tested conditions.

Collectively, these findings demonstrate that the OTU proteins from *M. larici-populina* and *T. deformans* are associated with reduced ubiquitination levels from host proteins, supporting their classification as functional deubiquitinases.

### 3.5. MlpOTU and TdOTU Do Not Induce Robust Apoptosis

**Figure 6:**
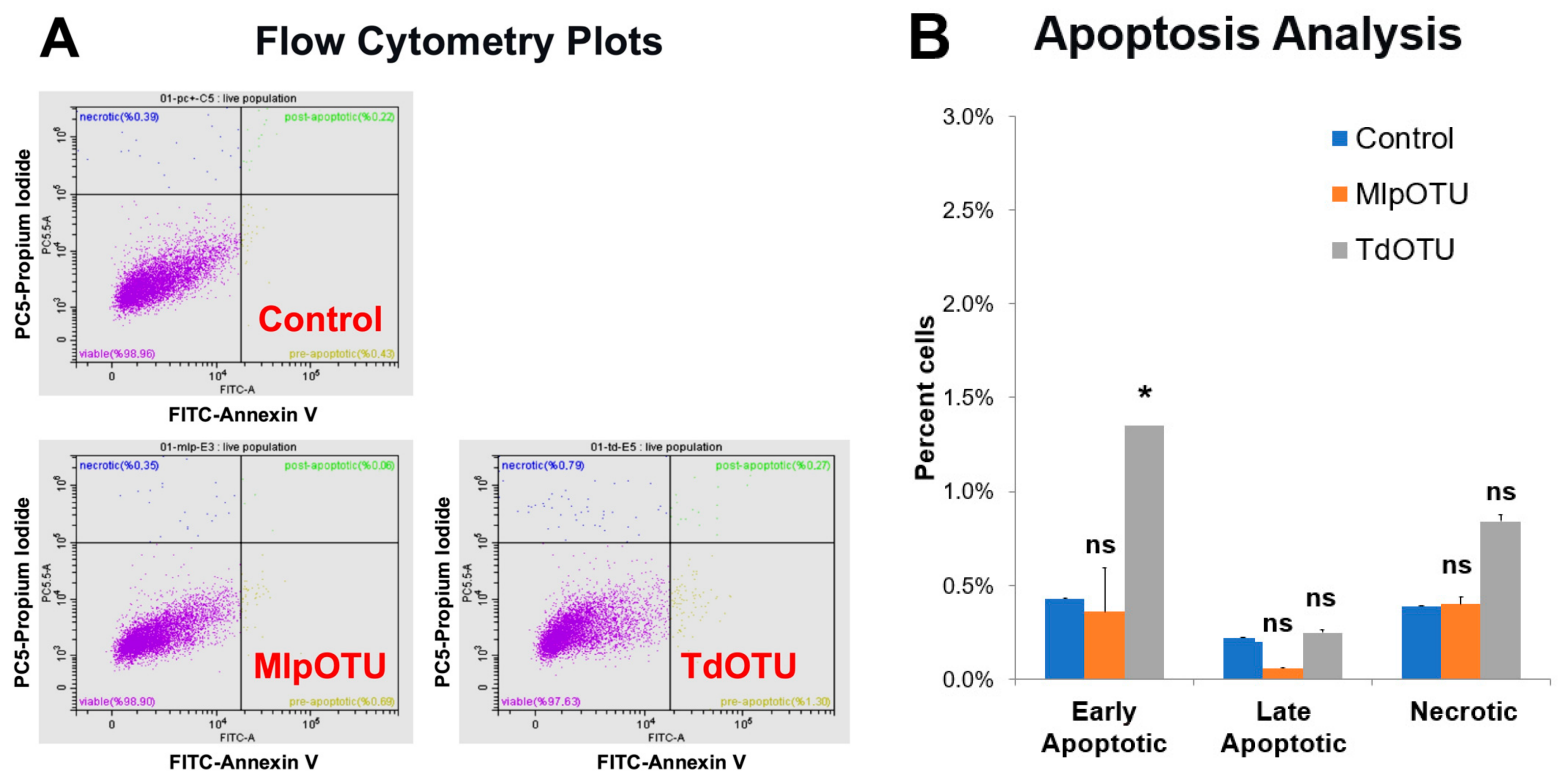
Apoptosis analysis by flow cytometry. (**A**) Representative dot plot of Annexin V-FITC/PI staining for HEK293T cells transfected with empty pcDNA3.1 vector (control), MlpOTU-transfected cells and TdOTU-transfected cells. (**B**) Quantification of pre-apoptotic, post-apoptotic, and necrotic cell percentages. No significant differences in apoptosis were observed between OTU-expressing cells and the empty vector control. Data are presented as mean ± SD. * *p* < 0.05. ns: not significant.

To assess whether MlpOTU or TdOTU expression triggers programmed cell death, we performed Annexin V-FITC/PI staining followed by flow cytometry (Figure 6A,B). Cells transfected with empty pcDNA3.1 vector showed 98.96% viability, with 0.43% early apoptotic, 0.22% late apoptotic, and 0.39% necrotic cells. MlpOTU-expressing cells showed 98.90% viability, 0.69% early apoptotic, 0.06% late apoptotic, and 0.35% necrotic cells. TdOTU-expressing cells showed 97.63% viability, 1.30% early apoptotic, 0.27% late apoptotic, and 0.79% necrotic cells. While TdOTU-expressing cells showed a slight increase in early apoptotic cells (1.30% vs. 0.43% for control, *p* = 0.036), overall viability remained above 97% and no overt induction of apoptosis was observed under these experimental conditions. These results indicate that neither MlpOTU nor TdOTU triggers significant programmed cell death in HEK293T cells.

## 4. Discussion

In this study, we provide the first functional characterization of putative OTU domain-containing proteins from two fungal plant pathogens, *Melampsora larici-populina* (MlpOTU) and *Taphrina deformans* (TdOTU). Our results demonstrate that both MlpOTU and TdOTU possess deubiquitinase activity, modulate host immune gene expression, and do not induce significant apoptosis, establishing them as potential virulence factors that may contribute to evasion.

The successful expression and purification of recombinant MlpOTU and TdOTU were achieved using IPTG induction in *E. coli*. IPTG, a non-metabolizable lactose analog, binds the lac repressor and derepresses the lac operon in pET vectors, enabling high-level protein production. After sonication and nickel-affinity chromatography (HisTrap HP), both proteins were obtained at high purity (>90%), as confirmed by SDS-PAGE. The use of imidazole for competitive elution efficiently released the His-tagged OTUs from the nickel column. Subsequent ultrafiltration with 30 kDa cut-off filters was intended to concentrate the proteins and remove smaller impurities. However, the final concentrations determined by BCA assay were relatively low (0.22 mg/mL for MlpOTU, 2.70 mg/mL for TdOTU). This may be explained by the molecular weights of MlpOTU (36 kDa) and TdOTU (38 kDa) being close to the filter cut-off, leading to partial loss during ultrafiltration.

Solubility analysis in PBS revealed that both proteins exhibited high solubility (>90%), with the supernatant containing properly folded protein and the pellet showing little to no visible band. Proper folding is essential for enzymatic function, as hydrophobic regions become buried and hydrophilic regions face the solvent. The high solubility thus indicates that the recombinant MlpOTU and TdOTU are likely in a functional state, suitable for structural studies and inhibitor screening.

The observation that both MlpOTU and TdOTU reduce global ubiquitination levels in HEK293T cells provides direct evidence that these fungal proteins possess functional deubiquitinase activity. Western blot analysis using an antibody that detects both mono- and poly-ubiquitinated proteins showed up to 50% reduction in ubiquitin signal, while an antibody specific for poly-ubiquitin chains revealed up to 70% decrease. These findings confirm that the OTU domains are associated with reduced ubiquitination in the host proteins.

The preferential effect on mono-ubiquitinated proteins and low-molecular-weight substrates is particularly interesting. Mono-ubiquitination regulates diverse cellular processes including endocytosis, histone regulation, and DNA repair [11]. Therefore, MlpOTU and TdOTU may target specific signaling pathways rather than globally affecting proteasomal degradation. This specificity could allow the fungi to fine-tune host immune responses without causing widespread proteolytic disruption.

The effects of MlpOTU and TdOTU on host immune gene expression were distinct and informative. MlpOTU produced a complex pattern that resembles the immune evasion strategy employed by viral OTU proteases. Notably, it increased the expression of *IL18* (a pyroptosis-associated cytokine) but decreased *IL1B*. This suggests that MlpOTU may interfere with inflammatory cytokine production while potentially triggering other arms of the pyroptosis pathway [12]. In the double-stranded DNA sensing pathway, *STING* and *TBK1* were unchanged, whereas *IRF3* was slightly downregulated, implying that MlpOTU may partially uncouple pathogen recognition from signal propagation [13]. Similarly, in the double-stranded RNA sensing pathway, *TLR3* and *RIG-I* were increased, but *MDA5*, *MAVS*, and *NFKB1* were decreased. This pattern indicates that MlpOTU may activate initial sensing while blocking the downstream transcriptional response.

The most striking finding was the severe suppression of *MX1* expression by MlpOTU, a dynamin-like *GTPase* with broad antiviral activity and a key effector of the type I interferon response [14,15]. As the suppression of *MX1* would create a more permissive environment for pathogen survival, it suggests that MlpOTU may specifically target interferon-stimulated gene expression to facilitate fungal infection [16]. In line with this, the potent suppression of *MX1* mirrors a known immune evasion strategy employed by certain intracellular parasites. Notably, while other ISGs like *APOBEC3G* and *G1P2* were increased, the selective suppression of *MX1* indicates that MlpOTU’s interference is specific rather than globally affecting the ISG response. This selective targeting could allow the pathogen to fine-tune the host environment for its survival without completely dismantling all antiviral defenses [17].

In stark contrast, TdOTU expression led to the overexpression of most tested immune genes, particularly a dramatic increase (20- to 100-fold) in type I interferons (*IFNA1, IFNA2*) and *MX1* itself. This pattern is the opposite of what would be expected for a classical immune-evasion protein. While this result may initially seem contradictory, several non-exclusive explanations must be considered. First, the observed transcriptional changes may reflect a cellular stress response or unfolded-protein response triggered by heterologous expression of a fungal DUB in human cells, rather than a specific immune-modulatory function. Second, host surveillance pathways could sense the deubiquitinase activity or the foreign protein itself and mount an antiviral-like transcriptional program. Third, TdOTU might interact with substrates or co-factors present in human cells that are absent in its natural plant host, leading to off-target signalling. Because *T. deformans* is a plant pathogen that has not been in contact with mammalian immune components, the gene-expression signature observed in HEK293T cells may be an artefact of the heterologous system. Importantly, certain fungal OTU deubiquitinases are essential for pathogenicity in their respective plant hosts [18,19], and it is possible that TdOTU performs a critical, context-dependent function in its native environment such as regulating fungal development or counteracting specific plant defence pathways that is not recapitulated in human cells. Therefore, while the current data do not support an immune-suppressive role for TdOTU in the human cell context, they also do not exclude a virulence function in plants. Future studies using plant infection models and stress-reporter assays will be required to determine whether the TdOTU-induced gene expression is a direct consequence of host immune modulation, an indirect effect of proteotoxic stress, or a combination thereof.

The finding that neither MlpOTU nor TdOTU induces significant apoptosis (Annexin V-FITC/PI staining) is consistent with their proposed role as evasion factors rather than cytopathic effectors. Cells expressing MlpOTU or TdOTU showed high viability (>97%) with only minor, statistically increases in early apoptotic cells. By avoiding host cell death, these fungal proteins may allow the pathogen to establish persistent infection within the host plant [20].

*T. deformans* has been shown to cause significant leaf hypertrophy and economic loss across numerous cultivars, underscoring the need to understand the molecular toolkit this pathogen uses to manipulate host growth and immunity [21]. While previous local studies have focused on biochemical responses like antioxidant enzyme activity and pigment changes, our characterization of TdOTU provides a novel perspective on how the pathogen might utilize deubiquitination to hijack host signaling pathways. Furthermore, the characterization of MlpOTU as a functional deubiquitinase aligns with emerging cross-kingdom research regarding the ovarian tumor domain family. Studies on OTU family proteins emphasize their dual role in regulating both tumor progression and immune escape, primarily through the modulation of *NF-kB* and cytokine signaling. By demonstrating that MlpOTU suppresses key immune effectors like *MX1* and *NFKB1*, our study confirms that fungal pathogens utilize the same conserved enzymatic strategies found in more widely studied viral and bacterial pathogens in the region. This suggests that the OTU domain is a universal tool for immune evasion, providing a basis for future collaborative efforts to develop broad-spectrum antifungal strategies within the academic and agricultural communities.

Several limitations of this study should be acknowledged. First, our functional analyses were performed in human HEK293T cells rather than in plant cells or fungal-infected tissues. While this allowed us to characterize enzymatic and immune-modulatory functions in a well-defined system, future studies should confirm these findings in the natural plant hosts (e.g., poplar for *M. larici-populina* and peach for *T. deformans*). Second, the specific host proteins targeted by MlpOTU and TdOTU remain unknown. Mass spectrometry-based ubiquitin remnant profiling could identify endogenous substrates. Third, the precise catalytic mechanisms and substrate preferences of these fungal OTU domains require further biochemical characterization using purified components (e.g., in vitro DUB assays with ubiquitin-AMC). Fourth, although our data suggest that MlpOTU and TdOTU are folded, direct measurement of enzymatic activity (e.g., using fluorogenic substrates) is needed to confirm their catalytic efficiency. Fifth, because *T. deformans* is a plant pathogen that has not been associated directly with mammalian immune components, the lack of immune suppression by TdOTU in HEK293T cells could simply reflect the absence of relevant plant-specific host targets. This still requires confirmation in plant infection models.

Despite these limitations, our study establishes MlpOTU and TdOTU as functional deubiquitinases with the ability to modulate host immune responses. These findings open new avenues for understanding fungal pathogenesis and may lead to the development of novel antifungal strategies targeting OTU domains. Future work should focus on solving the crystal structures of these OTU domains, screening for small-molecule inhibitors, and performing infection studies in plant models to validate their role in virulence.

In this study, we have demonstrated that both MlpOTU and TdOTU can be successfully expressed and purified as soluble, properly folded proteins with high purity, enabling their biochemical characterization. Functional analysis revealed that both OTU domains reduce global ubiquitination levels in mammalian cells, confirming their intrinsic deubiquitinase activity. Furthermore, these proteins modulate host innate immune gene expression; notably, MlpOTU exhibited a pattern consistent with evasion, including the selective suppression of the antiviral effector *MX1*. Importantly, neither MlpOTU nor TdOTU induced robust apoptosis in transfected cells, suggesting that they may facilitate pathogen survival without triggering host cell death. Collectively, these findings establish MlpOTU and TdOTU as functional deubiquitinases with the potential to contribute to fungal immune evasion. Future research should focus on identifying their specific host substrates, determining their three-dimensional structures, and developing small-molecule inhibitors that block their deubiquitinase activity. Additionally, functional studies in plant systems will be essential to understand the precise roles of these OTU domains in fungal pathogenesis and host immune evasion.

## Figures and Tables

**Table 1:** Properties of MlpOTU and TdOTU Inserts.

Property	MlpOTU	TdOTU
Species	*Melampsora larici-populina*	*Taphrina deformans*
Accession Number	EGG09943.1	CCG84064.1
Amino acids	330 aa	346 aa
Molecular Mass	36.02 kDa	38.41 kDa
Theoretical pI	5.9	5.26
GPI anchor	None	None
Predicted disulfide bonds	148–215; 301–304 (Number of Cysteines: 5)	167–251; 205–318; 236–315 (Number of Cysteines: 7)

## Data Availability

The original contributions presented in this study are included in the article. Further inquiries can be directed to the corresponding author.

## Supplementary Materials

The following supporting information can be downloaded at: https://www.mdpi.com/article/10.3390/jof12050361/s1. Table S1. Complete list of primers used for Q-PCR; Table S2. Concentration Calculation of MlpOTU and TdOTU; Table S3. Expected Band Sizes of Recombinant Plasmids.
