# Supplementary material for: Functional Characterization of OTU Domain-Containing Deubiquitinases from Plant Pathogenic Fungi Reveals Distinct Immune Modulatory Mechanisms
Source: J Fungi (Basel). 2026 May 14;12(5):361. doi: 10.3390/jof12050361 (PMC13208475; doi:10.3390/jof12050361)
Supplement: Supplementary file 1 [file jof-12-00361-s001.zip › jof-4286832-supplementary.pdf]

Supplementary Information

Table S1. Complete list of primers used for Q-PCR

Gene	Forward Primer (5'-3')	Reverse Primer (5'-3')
GAPDH	AATGAAGGGGTCATTGATGG	AAGGTGAAGGTCGGAGTCAA
STING	GATATCTGCGGCTGATCCTG	ATATACAGCCGCTGGCTCAC
TBK1	GCAGTTTGTTTCTCTGTATGGC	AATGTTACCCCAATGCTCCA
IRF3	ATGCACAGCAGGAGGATTTC	GTTGGCAGGTCTGGCTTATC
IL1B	GAAGCTGATGGCCCTAAACA	AAGCCCTTGCTGTAGTGGTG
IL18	TGCAGTCTACACAGCTTCGG	ACTGGTTCAGCAGCCATCTT
RIGI	ATATCCGGAAGACCCTGGAC	GAGAAAAAGTGTGGCAGCCT
MDA5	TTCAACCACAGTTCAGCCAA	TGACACTTCCTTCTGCCAAA
MAVS	GGTCGCCAGGTCTCAGG	TGTCTTCAGCAAACGGCAT
NFKB	ATGTATGTGAAGGCCCATCC	ATAACCTTTGCTGGTCCCAC
IFNA1	CAGAGTCACCCATCTCAGCA	CTTGACTTGCAGCTGAGCAC

IFNA2	GCTCACCCATTTCAACCAGT	CTTGACTTGCAGCTGAGCAC
IFNAR1	GACCCTAGTGCTCGTCGC	ACTCATCGCTCCTGTTCCAC
IFNB1	CTTTCGAAGCCTTTGCTCTG	CAGGAGAGCAATTTGGAGGA
STAT1	TTCAGGAAGACCCAATCCAG	TGCTCTGAATATTCCCCGAC
APOBEC3G	AGGGGCTTTCTATGCAACC	TTCCAAAAGGGAATCACGTC
G1P2	GCGAACTCATCTTTGCCAGT	AGGGACACCTGGAATTCGTT
IL15	AGAAGCCAACTGGGTGAATG	ACTTTGCAACTGGGGTGAAC
MX1	GATTTTGGGGCTTTCCAGTC	GATGATCAAAGGGATGTGGC
TLR3	AGGAAAGGCTAGCAGTCATCC	GCTGCAGTCAGCAACTTCAT

Table S2. Concentration Calculation of MlpOTU and TdOTU

Sample	Absorbance at 562 nm (blank subtracted)	Concentration (mg/mL)
MlpOTU	0.197	0.220
TdOTU	1.963	2.699

Table S3. Expected Band Sizes of Recombinant Plasmids

Construct	Vector size (bp)	Restriction enzymes	Expected bands (bp)
MlpOTU-pcDNA3.1(+)	6427	NdeI + EcoRI	431, 1056, 1486, 4960
TdOTU-pcDNA3.1(+)	6475	NdeI + EcoRI	431, 1104, 1535, 4960
